# Supplementary figures and images for: Establishment of transgenic fluorescent mice for labeling synapses and screening synaptogenic adhesion molecules
Source: eLife. 2024 Mar 7;13:e81884. doi: 10.7554/eLife.81884 (PMC10948142; doi:10.7554/eLife.81884)

## Slide 1
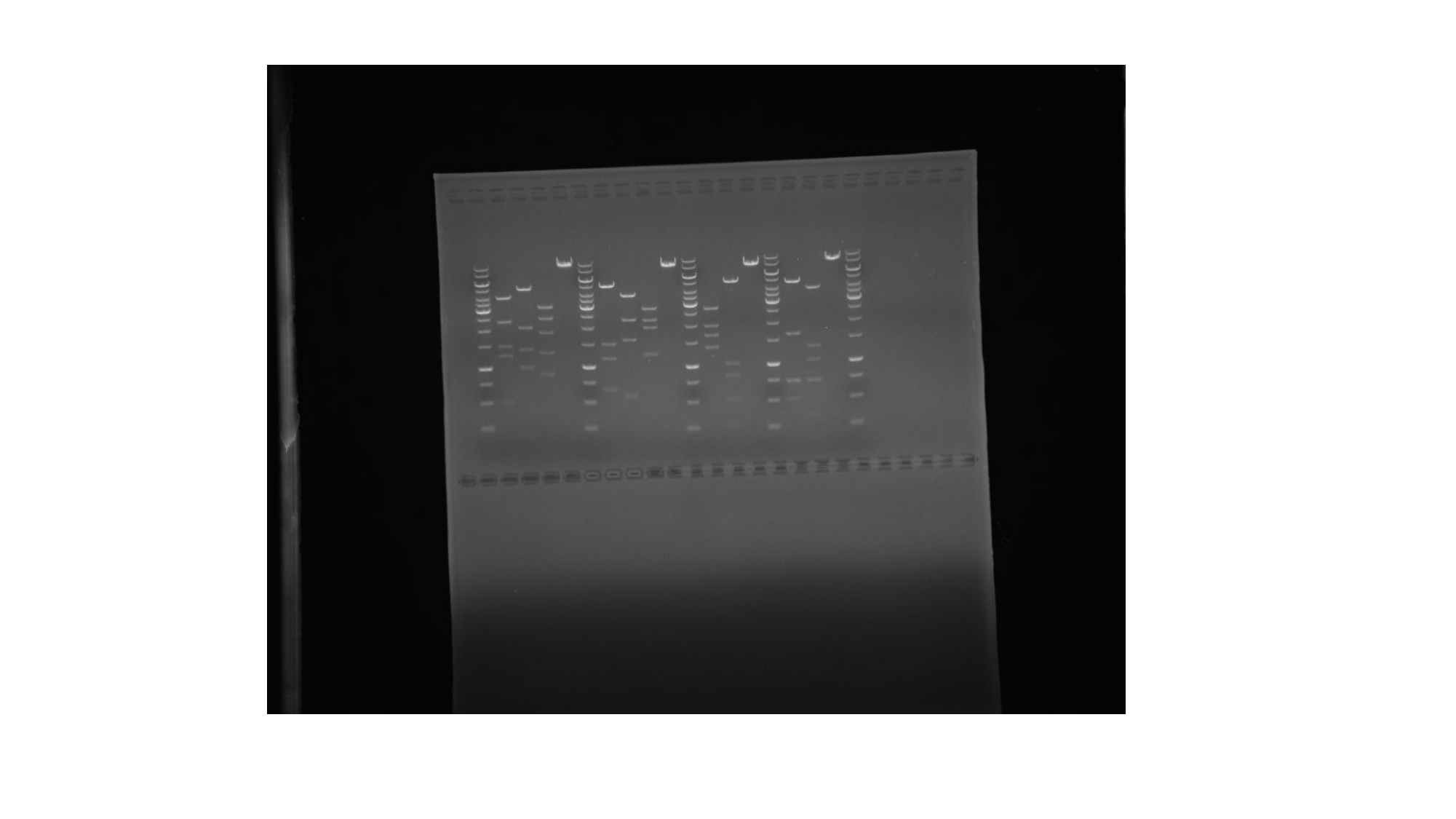

## Slide 2
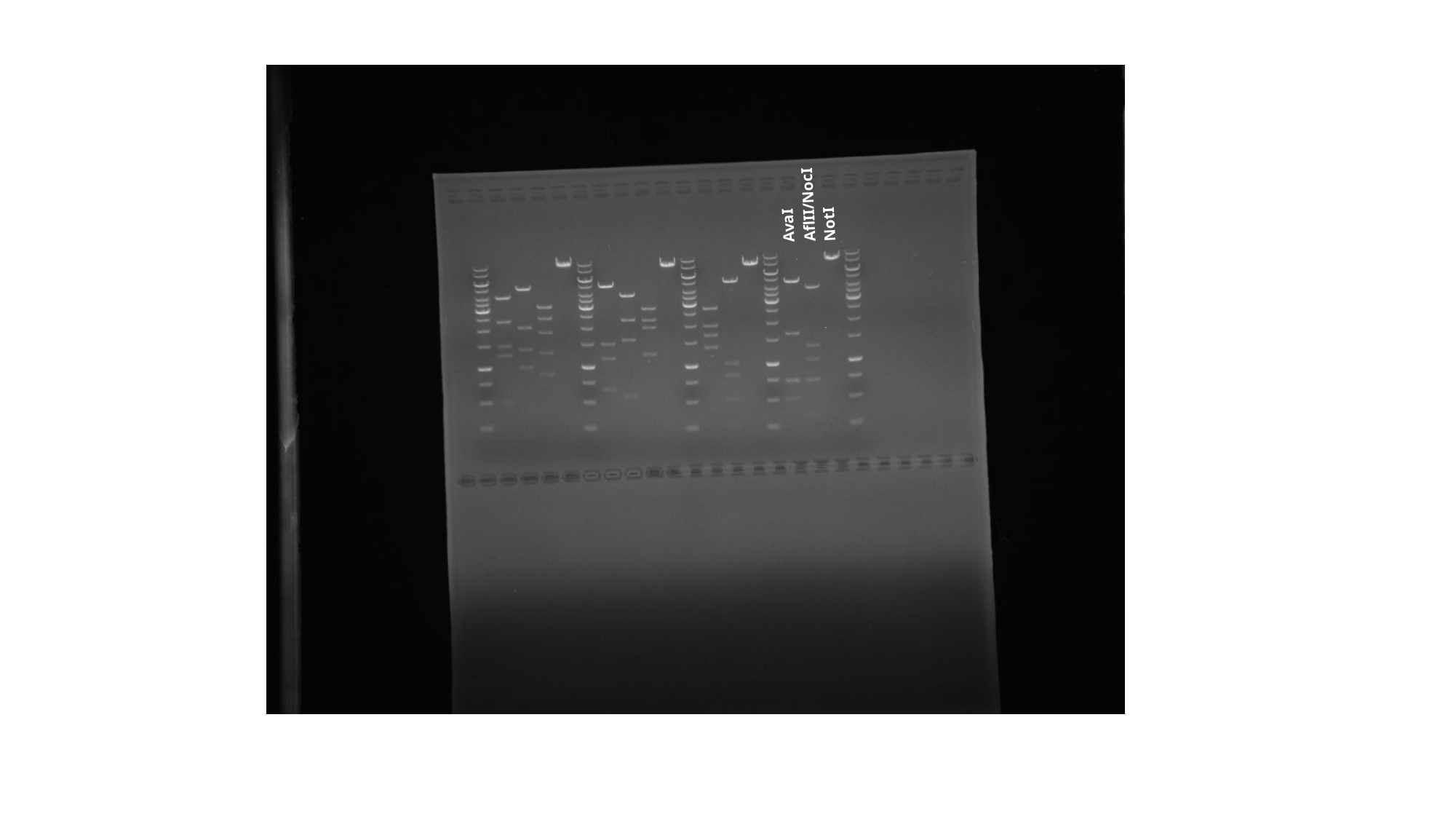

NotI
AflII/NocI
AvaI

## Slide 3
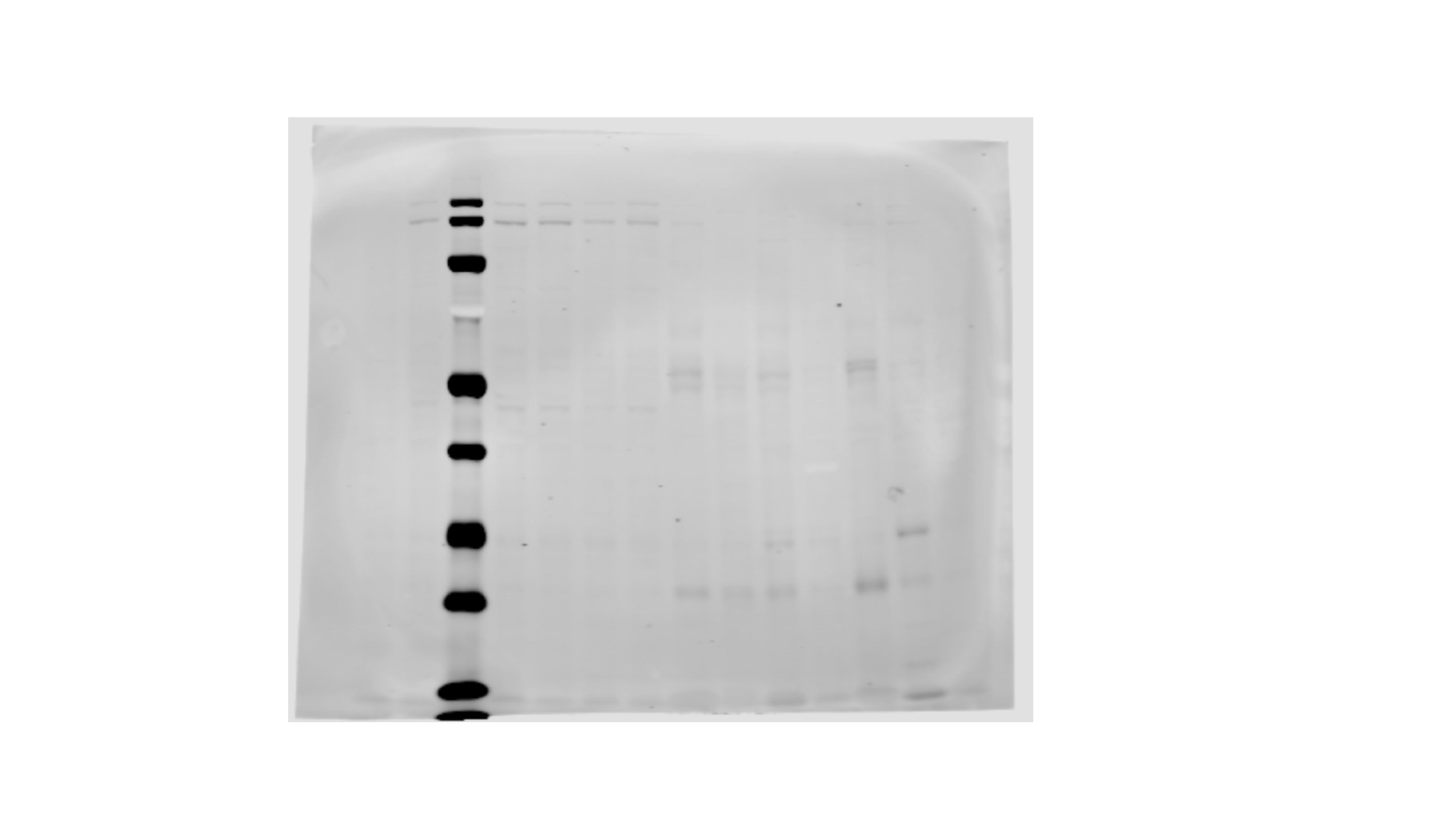

## Slide 4
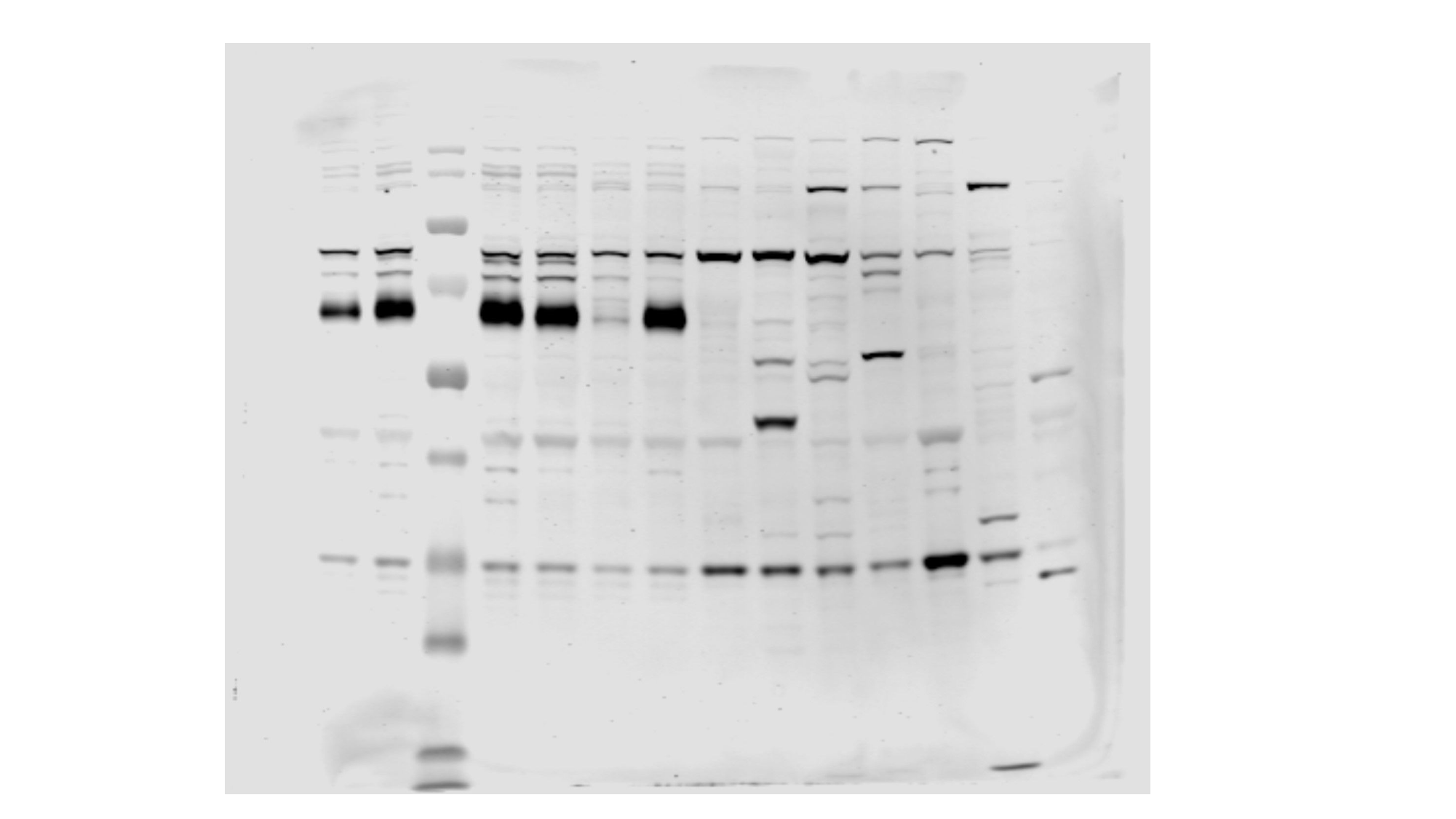

## Slide 5
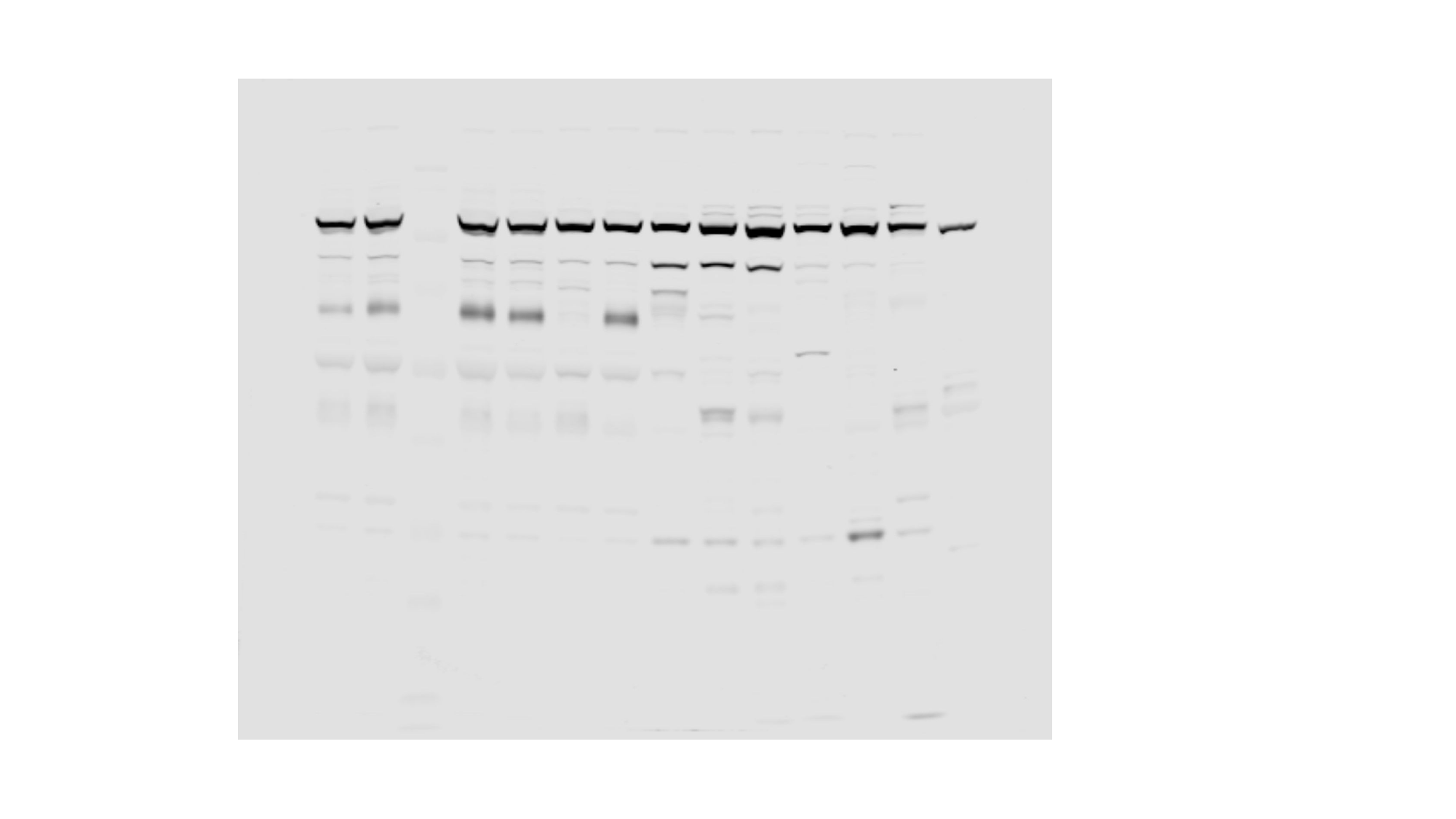

Supplement: Figure 1—source data 1. [file elife-81884-fig1-data1.zip › Figure 1/Figure 1-source data.pptx]
